# Genetically engineered human embryonic kidney cells as a novel vehicle for dual patch clamp study of human gap junction channels

**DOI:** 10.1042/BCJ20240016

**Published:** 2024-06-10

**Authors:** Honghong Chen, Yi X. Li, Robert S. Wong, Jessica L. Esseltine, Donglin Bai

**Affiliations:** 1 Department of Physiology and Pharmacology, University of Western Ontario, London, Ontario, Canada N6A 5C1; 2 Division of BioMedical Sciences, Faculty of Medicine, Memorial University of Newfoundland, St. John's, Newfoundland and Labrador, Canada A1B 3V6

**Keywords:** Cx43, gap junction channel, HEK-293 cells, patch clamp, single channel analysis

## Abstract

Mutations in more than half of human connexin genes encoding gap junction (GJ) subunits have been linked to inherited human diseases. Functional studies of human GJ channels are essential for revealing mechanistic insights into the etiology of disease-linked connexin mutants. However, the commonly used *Xenopus* oocytes, N2A, HeLa, and other model cells for recombinant expression of human connexins have different and significant limitations. Here we developed a human cell line (HEK293) with each of the endogenous connexins (Cx43 and Cx45) knocked out using the CRISPR-Cas9 system. Double knockout HEK293 cells showed no background GJ coupling, were easily transfected with several human connexin genes (such as those encoding Cx46, Cx50, Cx37, Cx45, Cx26, and Cx36) which successfully formed functional GJs and were readily accessible for dual patch clamp analysis. Single knockout Cx43 or Cx45 HEK cell lines could also be used to characterize human GJ channels formed by Cx45 or Cx43, respectively, with an expression level suitable for studying macroscopic and single channel GJ channel properties. A cardiac arrhythmia linked Cx45 mutant R184G failed to form functional GJs in DKO HEK293 cells with impaired localizations. These genetically engineered HEK293 cells are well suited for patch clamp study of human GJ channels.

## Introduction

Gap junction (GJ) channels are ubiquitous in our body mediating direct intercellular communications in various tissues and organs [1,2]. A GJ channel is composed of two head-to-head docked hemichannels and each hemichannel is a hexamer of connexins [2]. Twenty-one connexin genes are in the human genome encoding different connexins with distinct tissue distribution [3,4]. Each tissue cell often expresses more than one type of connexin to form various types of GJ channels to meet the demand of intercellular communication in different tissues and organs [1,5]. The biological importance of GJ channels is best demonstrated by the fact that numerous mutations in more than half of connexin genes have been linked to inherited diseases, including sensorineural hearing loss, hypomyelination diseases, cataracts, cardiac arrhythmias, and developmental disease [6–8]. To establish if these disease-linked connexin variants play a pathogenic role in patients carrying connexin variant genes, one would need to have evidence of functional impairment associated with these variants. However, most of the functional studies on human disease-linked connexin mutants are conducted on non-mammalian *Xenopus* oocyte expression systems, rodent cell lines, such as mouse neuroblastoma cells (N2A), and/or a human cervical cancer cell lines, HeLa cells [9–14]. In these expression systems, human connexins were mostly expressed well and were able to assemble into functional hemichannels and/or GJ channels for functional characterization. However, each of these commonly used expression systems showed some important limitations.

*Xenopus* oocytes are excellent cells for recombinant expression of connexins/innexins from vertebrates and invertebrates for their GJ and/or hemichannel studies [15–17]. The large size of the oocytes and their nuclei made it possible to inject mRNA or cDNA encoding for connexin genes and study the resultant GJ or hemichannel properties with two-electrode voltage clamp technique [18,19]. This approach is still used by several laboratories to study GJ and/or hemichannels [9,13,20]. But here are some limitations for functional studies of GJ channels using *Xenopus* oocytes: (1) they are non-mammalian cells with different posttranslational modifications from those in the mammalian cells, such as, phosphorylation, glycosylation, ubiquitination, etc. [21–23]; (2) to study GJ functions in oocyte pairs requires dedicated specialized two-electrode voltage clamp systems to perform voltage clamp [18,19]; (3) endogenously expressed Cx38 could form functional GJs or mix with expressed connexins to form various types of GJs, complicating data analysis and interpretations [16,24]. This was a big issue in early studies prior to the use of antisense RNA against Cx38; (4) the number of GJ channels formed between oocyte pairs is very high and therefore this approach cannot be used for single GJ channel studies.

Mouse neuroblastoma (N2A) cells are mammalian cells with many posttranslational modifications closely resemble to those in human cells and they are well suited to study both macroscopic and single GJ channel currents with dual patch clamp technique [25–27]. N2A cells are connexin-deficient, susceptible to transfection of connexin genes, and their sphere shape makes them the best choice for patch clamp study of human GJs. Though most human connexins are expressed well in N2A cells and able to form functional GJs [28–30], human Cx46 has been shown to be extremely difficult to express in this mouse cell line [11,31]. Researchers frequently use rodent version of Cx46 to study their GJ function [32,33]. The reason for human Cx46 not expressing well in N2A cells is not clear but may be related to the fact that this is a mouse cell line and may not represent the cellular context of human cells [26].

Human cervical cancer cell line, HeLa cell, is another commonly used model cell for GJ studies [34,35]. Like those found in N2A cells, HeLa cells are also connexin deficient and susceptible to transfection of connexin genes or generating stable connexin expression lines. But their flat morphology is not a friendly feature for patch clamp study for GJ or hemichannels. This feature, however, is more suitable for morphological studies on GJ localizations [10,30,32]. In addition, HeLa cells are also frequently used for dye transfer through GJ channels or dye upload via connexin hemichannels or other large pore channels [36,37]. One of the biggest limitations of using HeLa cells for functional study of GJ channels is its high genetic instability [38], which could change their morphology and, in some cases, turn on expression of connexins to form background GJ coupling contaminating the expressed GJ channels [39].

There are other less commonly used expression model cells, such as (1) human hepatoma SKHep1 cell line [40], which showed a relatively high (∼15%) background GJ coupling in cell pairs [41]; (2) rat insulinoma (RIN) cells were connexin deficient and were used for expression study of connexins [42–44] to study GJ properties and cell cycles. As RIN cells are rat cell line, it is not clear if all human connexins could express well and form functional GJs in this expression system. Even if they were expressed, they would still be in the cell context of a rodent cell line.

The limitations of these recombinant expression systems call for a novel improved *in vitro* model cell system to study human connexins, ideally in a human cellular context. Here we developed genetically engineered human embryonic kidney (HEK293) cells to eliminate endogenous connexins, by knocking out both Cx43 and Cx45 using CRISPR-Cas9. Double knockout HEK293 cells showed no GJ coupling for multiple passages, and were susceptible to transfection of human connexin genes, including Cx46, to form functional GJ channels. Importantly, these cells were easily accessible for dual patch clamp analysis for functional study of disease-linked connexin variants. We also characterized GJ channel properties in single Cx43 or Cx45 knockout cells and confirmed that the GJs in these single knockout lines are formed by Cx45 or Cx43, respectively. The endogenously expressed Cx43 or Cx45 were expressed at a suitable level to study either macroscopic or single GJ channels. Taken together, genetically engineered HEK293 cells are excellent model cells for studying human GJ channels.

## Results

### Endogenous GJ coupling in HEK293 cells was eliminated in the cells with both Cx43 and Cx45 genes knocked out

**Figure 1.**
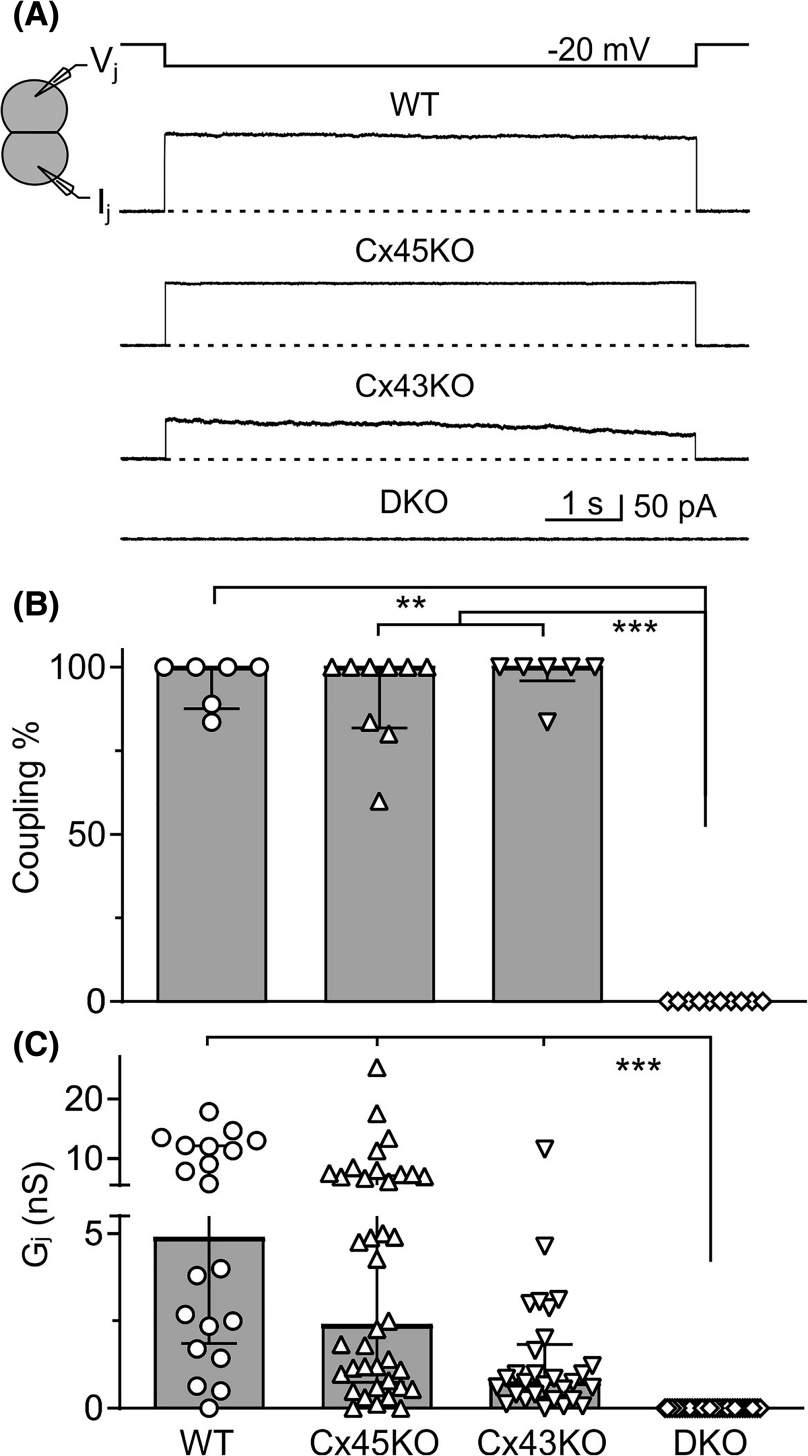
Coupling status of genetically engineered HEK293 cells. (**A**) Dual whole cell patch clamp technique was used to measure transjunctional current (*I_j_*) in response to indicated *V_j_* in HEK293 cell pairs. Representative *I_j_* recordings obtained from wildtype (WT), Cx45 knockout (Cx45KO), Cx43 knockout (Cx43KO), and double knockout with both Cx45 and Cx43 ablated (DKO) HEK293 cell pairs. (**B**) Bar graphs summarize the median percentage (the error bars indicate IQR) of coupled cell pairs (coupling%) in different HEK293 cells. Data points represent the number of transfections. (**C**) Bar graph illustrates the median coupling conductance (*G_j_*) of HEK293 cell pairs. Data points represent the number of cell pairs. Kruskal–Wallis test followed by Dunn's *post hoc* test was used to compare different groups for all bar graphs. The statistical significance was indicated (**P < 0.01, and ***P < 0.001).

To study endogenous GJ coupling in HEK293 cells, we selected isolated cell pairs to perform dual whole cell voltage clamp to measure transjunctional currents (*I_j_*s) in response to a transjunctional voltage (*V_j_* = −20 mV). As shown in Figure 1, representative *I_j_*s were recorded from WT, Cx45 knockout (Cx45KO), Cx43 knockout (Cx43KO) HEK293 cell pairs, but not in cell pair with both Cx43 and Cx45 knocked out (double knockout, DKO). The median percentage of recorded cell pairs showed GJ coupling (coupling%, Figure 1B) was calculated across several experiments for WT HEK293 (WT, 100 (88–100)%, *N* = 6), Cx45KO (100 (82–100)%, *N* = 9), Cx43KO (100 (96–100)%, *N* = 6), and DKO cell pairs (0 (0–0)%, *N* = 10). The coupling conductance (*G_j_*) was calculated from each individual cell pair tested with median *G_j_*, (4.9 (1.9–12.2) nS, *n* = 20) for WT, (2.4 (0.7–7.1) nS, *n* = 38) for Cx45KO, (0.7 (0.3–1.8) nS, *n* = 29) for Cx43KO, (0 (0–0) nS, *n* = 39) for DKO (Figure 1C). Both coupling% and *G_j_* were significantly reduced for DKO cells compared with WT, Cx45KO, and Cx43KO cells (Figure 1).

**Figure 2.**
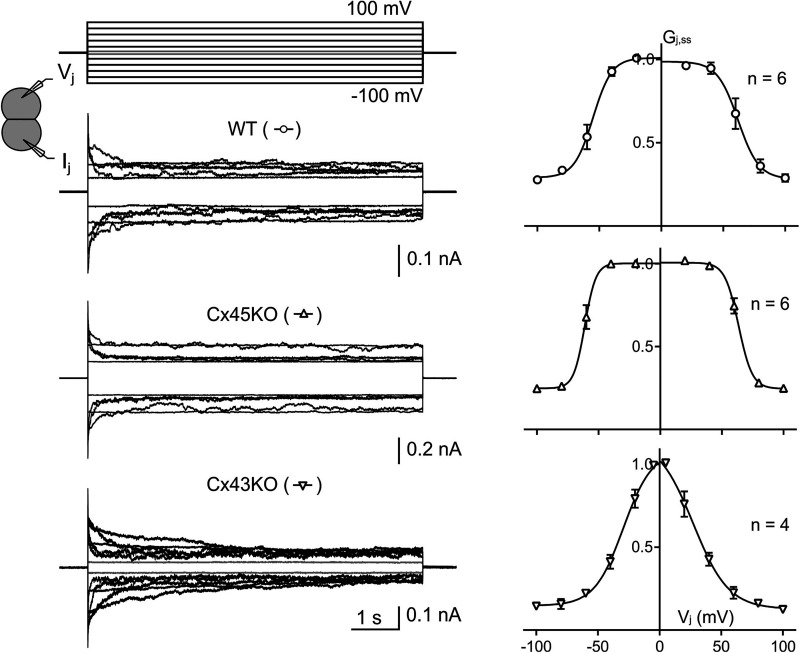
V_j_-gating properties of genetic engineered HEK293 cells. Superimposed transjunctional currents (*I_j_*s) in response to the indicated *V_j_* pulses (±20 to ±100 mV, in the case of Cx43KO ±5 mV *V_j_* pulses were also added and in gray color) for cell pairs of WT (open circles), Cx45KO (open triangles), and Cx43KO (open inverse triangles). Depending on the cells recorded, the absolute *I_j_* amplitude values were either remained steady during the *V_j_* pulse (at *V_j_*s of ±20 to ±40 mV for WT and Cx45KO cells and at *V_j_* of ±5 mV for Cx43KO cells) or decreased mirror symmetrically during the *V_j_* pulses (at the rest of the tested *V_j_*s). Normalized steady-state conductance (*G_j_*_,*ss*_) was obtained and plotted against tested *V_j_*s (right panel). The *G_j_*_,*ss*_ – *V_j_* relationship could be fitted well by Boltzmann equations and the smooth lines in each plot represent Boltzmann fittings for both *V_j_* polarities. The error bars represent the standard error of mean (±SEM). The number of cells pairs is indicated.

### *V_j_*-gating properties of endogenous GJs in engineered HEK293 cells

To investigate *V_j_*-gating properties of GJs in genetically engineered HEK293 cells, WT, Cx45KO, or Cx43KO cell pairs were selected for dual whole cell voltage clamp. *I_j_*s were recorded in response to a series of *V_j_* pulses (±20 to ±100 mV). As shown in Figure 2, *I_j_*s of WT, Cx45KO, and Cx43KO, all showed mirror symmetrical *V_j_*-dependent *I_j_* deactivation. For GJs of WT and Cx45KO cells, the *I_j_* deactivation was prominent when absolute *V_j_* values were ≥60 mV, while the Cx43KO cells showed *I_j_* deactivation when the absolute *V_j_* values were ≥20 mV (Figure 2). To obtain *V_j_* induced *I_j_*s with minimum *V_j_*-gating for Cx43KO cells, we added an additional pair of *V_j_* at ±5 mV, which indeed showed minimum *V_j_*-dependent deactivation (Figure 2 gray colored *V_j_*s and *I_j_*s). The steady-state conductance was normalized to the initial peak conductance for each *V_j_* pulse to obtain the normalized steady state conductance (*G_j_*_,*ss*_). The *G_j_*_,*ss*_ was then plotted against *V_j_* for each of these engineered HEK293 cells as shown in Figure 2 right panels. The *G_j_*_,*ss*_ – *V_j_* plots could be fitted well with Boltzmann equations for both *V_j_*-polarities for WT, Cx45KO, and Cx43KO cells (smooth lines in right panels of Figure 2 and Table 1 for Boltzmann parameters).

**Figure 3.**
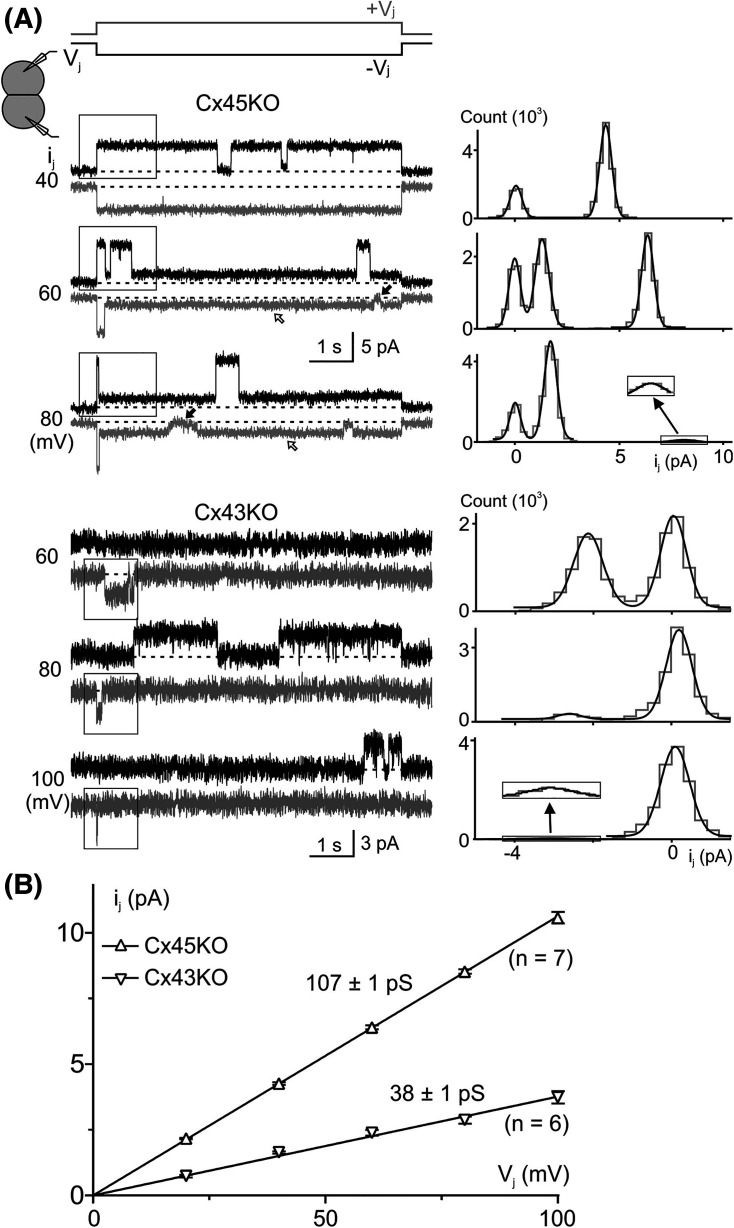
Single channel analysis of genetically engineered HEK293 cells. (**A**) Single channel currents (*i_j_*s) recordings were shown in response to the indicated *V_j_*s (−*V_j_* in black and +*V_j_* in gray) from Cx45KO (top) and Cx43KO (bottom) cell pairs. The Cx45KO *i_j_*s showed subconductance state (or residue conductance state, open arrows) and fully closed state (filled arrows). All point histograms (gray) were constructed in the boxed regions on the original *i_j_*s shown on the left panels and Gaussian fitting curves (black smooth curves) were used to measure the amplitude of the *i_j_*s at the main open state. (**B**) The *i_j_*s from Cx45KO (open triangles) and Cx43KO (inverse open triangles) were plotted against *V_j_*s. Linear regressions were used to determine the slope single channel conductance (*γ_j_*) for Cx45KO and Cx43KO cell pairs. The *γ_j_*s and the number of cell pairs are indicated.

**Table 1.** Boltzmann fitting parameters of functional homotypic and heterotypic gap junction channels

Cells	*V_j_* polarity	*G*_min_	*V*_0_ (mV)^†^	*A*
WT (*n* = 6)	+	0.28 ± 0.05	62.0 ± 2.6	0.12 ± 0.04
	−	0.29 ± 0.03	55.2 ± 1.8	0.13 ± 0.03
Cx45KO (*n* = 6)	+	0.25 ± 0.02	63.6 ± 1.1*	0.18 ± 0.04
	−	0.25 ± 0.03	61.3 ± 1.2***	0.22 ± 0.15
Cx43KO (*n* = 4)	+	0.13 ± 0.03*	27.2 ± 4.0***	0.07 ± 0.015
	−	0.15 ± 0.02*	29.4 ± 2.3***	0.085 ± 0.014

Data in this table are the average ± SEM. Boltzmann parameters of WT, Cx45KO, and Cx43KO HEK293 cells are shown. Statistically significant differences are indicated (*P < 0.05; ***P < 0.001) versus the WT parameters at the respective *V_j_* polarity; ^†^the absolute values of *V*_0_ are shown for each of the *V_j_* polarities.

### Unitary channel currents of engineered HEK293 cells

Cx45KO and Cx43KO HEK293 cell pairs sometimes showed only one functional GJ channel which allowed us to study single channel currents (*i_j_*s) at different *V_j_*s (Figure 3). All point histograms and Gaussian fits were used to measure the amplitudes of *i_j_*s for the main conducting state (the main open state) (Figure 3A, right panels). For Cx45KO cell pairs, one or more subconductance states (open arrows, also known as residue conductance states) as well as fully closed state (filled black arrows) were also observed in the recorded *i_j_*s (Figure 3A), while for Cx43KO cell pairs, only main open state could be identified. We constructed *i_j_* – *V_j_* plot for Cx45KO and Cx43KO cell pairs (Figure 3B). Linear regressions for the *i_j_* – *V_j_* plots were used to obtain the averaged slope single channel conductance for Cx45KO *γ_j_* = 107 ± 1 pS and for Cx43KO *γ_j_* = 38 ± 1 pS (Figure 3B).

**Figure 4.**
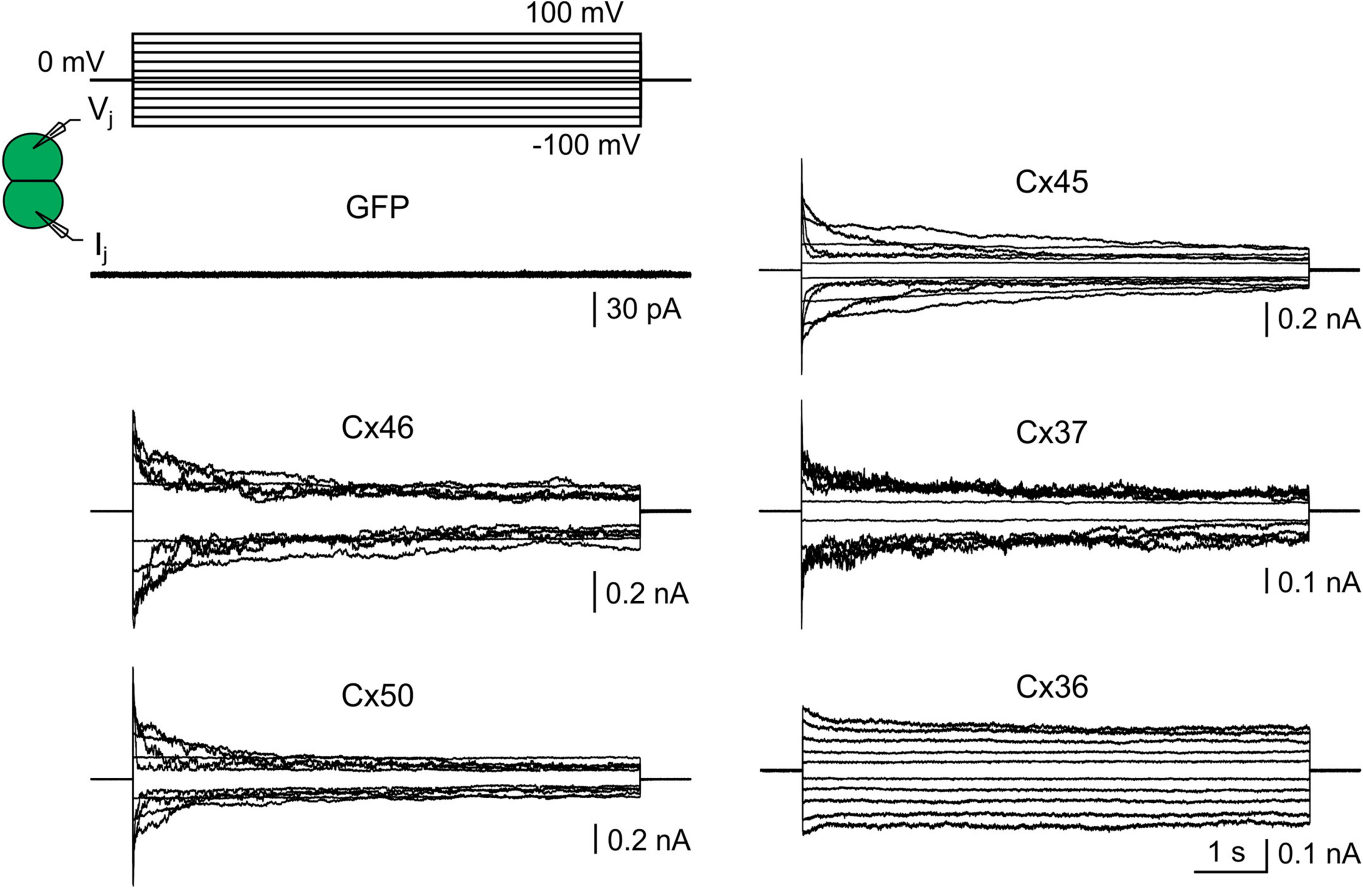
Expression of human connexins formed functional GJs in DKO HEK293 cells with signature *V_j_*-gating properties. Superimposed transjunctional currents (*I_j_*s) in response to the indicated *V_j_* pulses (±20 to ±100 mV, in some cases also with ±5 mV) for DKO cell pairs expressing GFP, Cx46, Cx50, Cx45, Cx37, or Cx36 as indicated. Expression of GFP alone failed to form any functional GJ channels, while expression of Cx46, Cx50, Cx45, Cx37, or Cx36 formed functional GJs with their signature *V_j_*-gating properties.

### Expression of several human connexins successfully formed functional GJs in double knockout HEK293 cells

**Figure 5.**
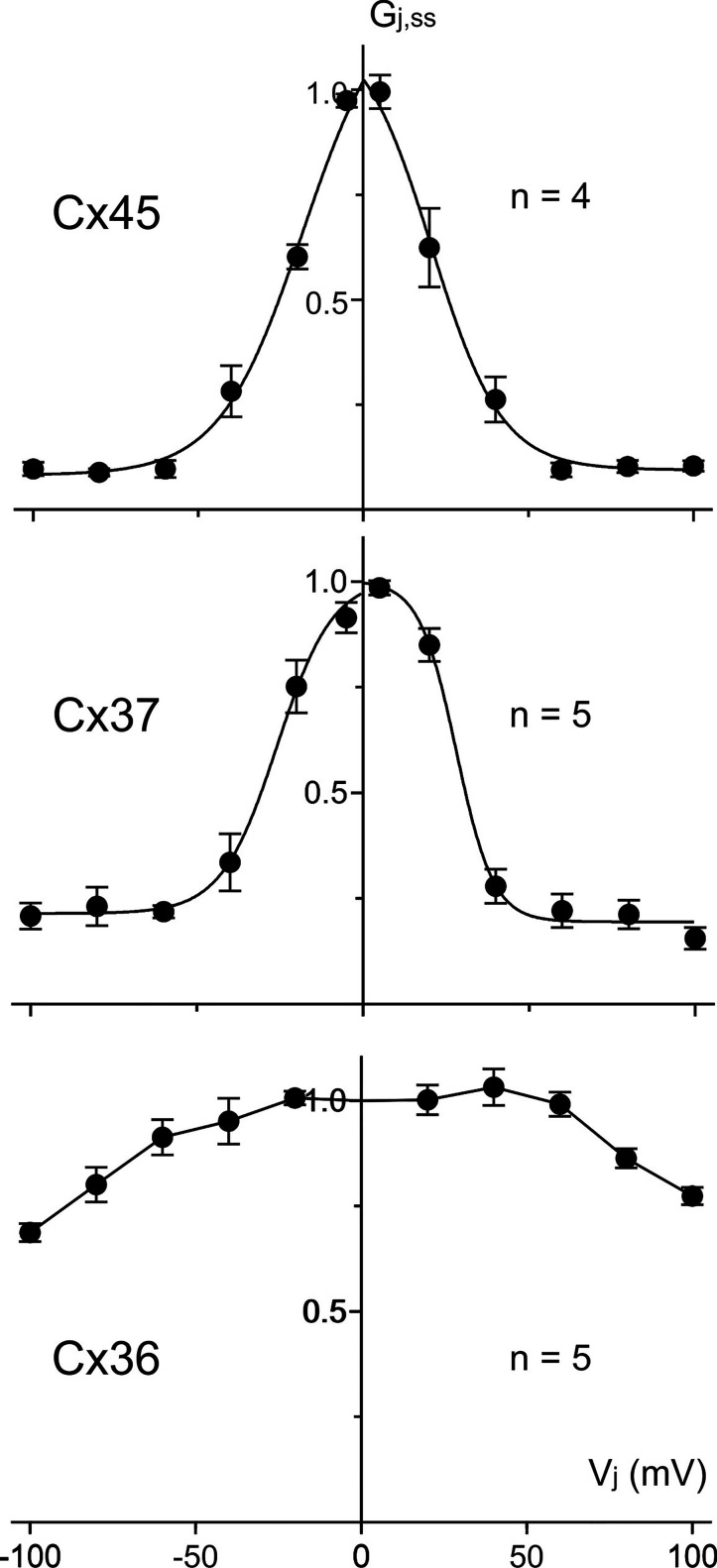
Normalized steady-state junctional conductance (*G_j_*_,*ss*_) was plotted against *V_j_* in cell pairs expressing Cx45, Cx37, and Cx36. The *G_j_*_,*ss*_ – *V_j_* plots of both Cx45 and Cx37 could be well fitted by Boltzmann equations for each of their *V_j_*s, while in the case of Cx36 we were unable to fit the data with Boltzmann equations (the line was simply connecting the averaged data points). The number of cell pairs are indicated for each plot.

To test if double knockout HEK293 cells (DKO) could be used to study recombinantly expressed human connexins, we transfected DKO cells with an expression vector containing human Cx45 (Cx45-IRES-GFP) and functional GJ channels were formed with *V_j_*-gating profile nearly identical with those observed in Cx43KO HEK293 cells (Figure 4). While expressing the empty vector (only encoding reporter GFP) as a negative control resulted in no GJ coupling (Figure 4). Similarly, we were able to express several selected human connexins, Cx46, Cx50, Cx37, and Cx36, in the DKO cells which successfully formed homotypic GJs with their signature *V_j_*-gating properties (Figure 4). Normalized steady-state junctional conductance (*G_j_*_,*ss*_) was plotted against *V_j_* (Figure 5). The *G_j_*_,*ss*_ – *V_j_* plots of cell pairs expressing Cx45 and Cx37 could be well fitted by Boltzmann equations (Figure 5 and Table 2). The Boltzmann parameters of these and our previously studied Cx46 and Cx50 GJs were very much similar to those obtained from the same connexins expressed in N2A cells, with the exception of the *G*_min_ of Cx37 GJ, which was consistently reduced for both positive and negative *V_j_* polarities (Table 2). The exogenously expressed Cx45 GJ *V_j_*-gating properties were also similar to those of endogenously expressed Cx45 (see Table 1 for Cx43KO). These data demonstrated that DKO HEK293 cells are an excellent model cell for patch clamp study of human GJs formed by these human connexins.

**Figure 6.**
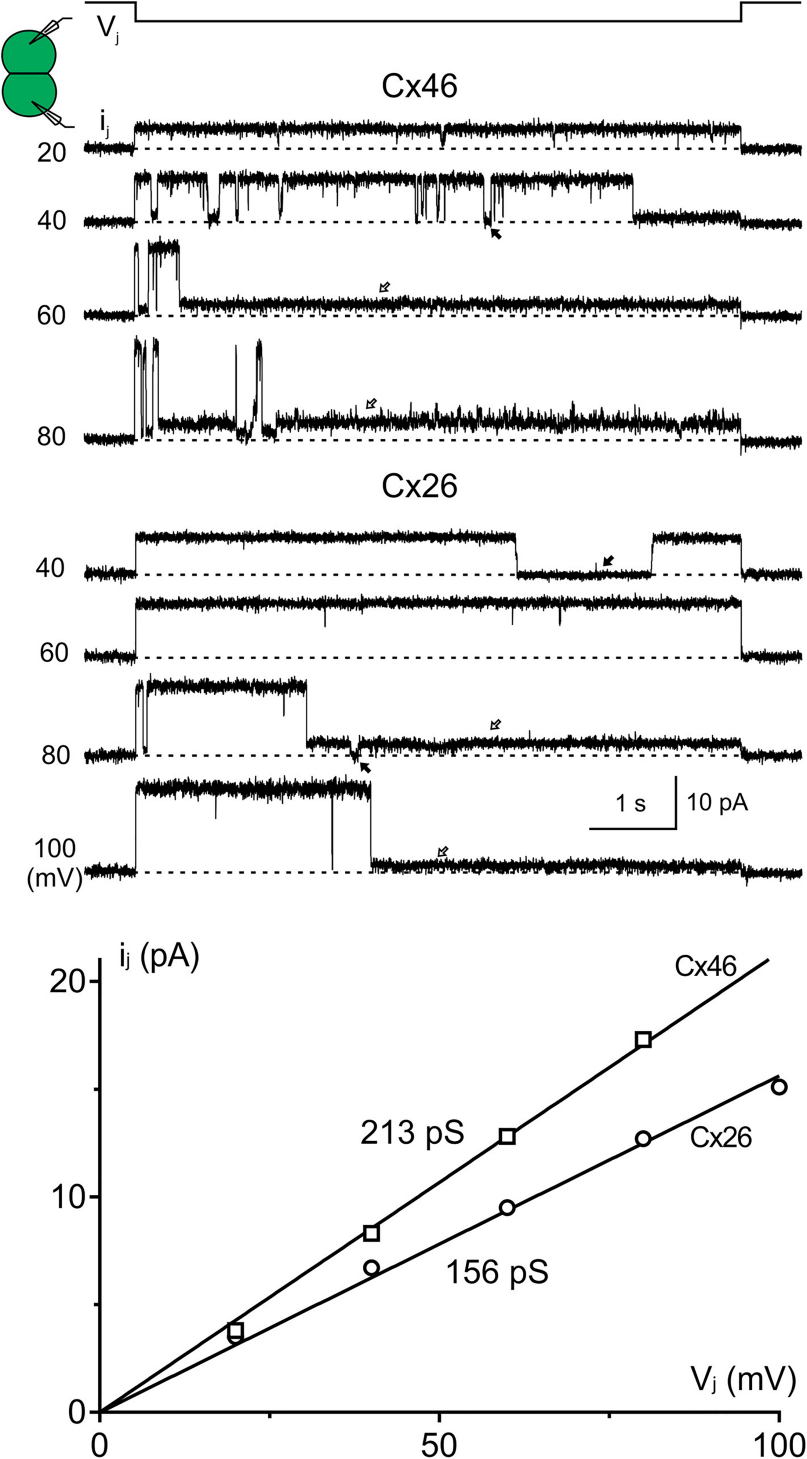
Single Cx46 and Cx26 GJ currents could be studied in DKO HEK293 cells. Top panel shows the unitary transjunctional currents (*i_j_*s) recorded in response to the indicated *V_j_* pulses in a DKO HEK293 cell pair expressing Cx46-IRES-GFP and Cx26-IRES-GFP. The amplitude of *i_j_*s increased with the increase of *V_j_* in each case and the linear regression of the *i_j_* – *V_j_* plot (bottom panel) for each of these cell pairs was used to obtain slope single channel conductance of Cx46 GJ (*γ_j_* = 213 pS) and Cx26 GJ (*γ_j_* = 156 pS). Open arrows on the *i_j_*s indicate residual conductance state, and the black filled arrow indicates fully closed state.

**Table 2.** Boltzmann fitting parameters of functional homotypic GJs expressed in DKO HEK293 cells

GJs	*V_j_* polarity	*G*_min_	*V*_0_ (mV)	*A*
Cx46 (*n* = 5)	+	0.10 ± 0.02	32 ± 1.7	0.18 ± 0.03
	−	0.16 ± 0.02	36.6 ± 2.1	0.21 ± 0.1
Cx50 (*n* = 4)	+	0.17 ± 0.02	35.5 ± 1.2	0.17 ± 0.04
	−	0.21 ± 0.02	37.5 ± 1.6	0.18 ± 0.07
Cx45 (*n* = 4)	+	0.08 ± 0.02	18.4 ± 3.7	0.08 ± 0.02
	−	0.09 ± 0.03	20.6 ± 3.6	0.09 ± 0.02
Cx37 (*n* = 5)	+	0.22 ± 0.03*	25.8 ± 2.5*	0.12 ± 0.03
	−	0.20 ± 0.02**	28.1 ± 1.5	0.18 ± 0.03

Data in this table are the average ± SEM. Boltzmann parameters of the indicated homotypic GJs are shown. Cx46 and Cx50 Boltzmann parameters are the same as reported previously [47]. Boltzmann parameters for Cx45 and Cx37 were compared with previous data [10,29] obtained for the same connexins expressed in N2A cells. Statistically significant differences are shown (*P < 0.05; **P < 0.001) versus the same connexin at the indicated *V_j_* polarity. *V*_0_ are reported as absolute values. No data were shown for Cx36, as the *G_j_*_,*ss*_ – *V_j_* plot for Cx36 GJ could not be well fit by the Boltzmann equation.

In poorly coupled DKO HEK293 cell pairs expressing Cx46 and Cx26, unitary channel currents (*i_j_*s) could be recorded, which is very useful in detailed mechanistic studies at a single GJ channel level with excellent signal noise ratio (Figure 6). The *i_j_* – *V_j_* plot was constructed for each of these cell pairs and the slope single channel conductance (*γ_j_*) for Cx46 (213 pS) and Cx26 (156 pS) was obtained (Figure 6).

**Figure 7.**
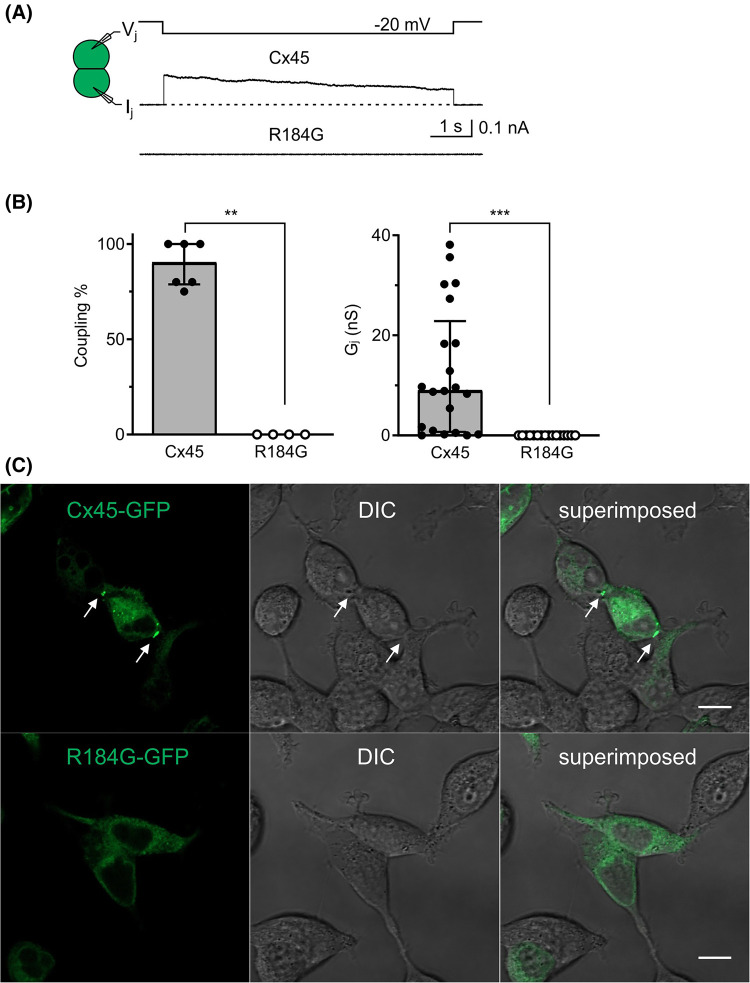
Cx45 R184G failed to form functional GJs in DKO HEK293 cells likely due to failed clustering at the cell-cell junctions. (**A**) Dual whole cell patch clamp technique was used to measure transjunctional current (*I_j_*) in response to indicated *V_j_* in DKO HEK293 cell pairs. Representative *I_j_* recordings obtained from cell pairs expressing untagged Cx45 or Cx45 R184G. (**B**) Bar graph on the left panel summarizes the median percentage (the error bars indicate IQR) of coupled cell pairs (coupling%) in cell pairs expressing Cx45 (filled circles) or Cx45 R184G (open circles). Data points represent the number of transfections. Bar graph on the right panel illustrates the median coupling conductance (*G_j_*) of cell pairs expressing Cx45 and Cx45 R184G. Data points represent the number of cell pairs. Mann–Whitney test was used to compare different groups for these bar graphs. The statistical significance was indicated (**P < 0.01, and ***P < 0.001). (**C**) Confocal images of DKO HEK293 cells expressing GFP tagged Cx45 (Cx45-GFP) or Cx45-R184G (R184G-GFP). GJ plaque like clusters of Cx45-GFP could be observed at the cell-cell junctions, whereas R184G-GFP failed to form any clusters at the cell-cell junctions. Fluorescence, differential interference contrast (DIC), and superimposed images are shown as indicated. These are representative images from three independent experiments. Scale bar = 10 μm.

### Human congenital heart disease and arrhythmia linked Cx45 variant R184G failed to form morphological and functional GJs

To reveal the mechanism of a congenital heart disease and arrhythmia linked Cx45 variant R184G [45], we expressed this variant in DKO HEK293 cells in two expression vectors, i.e. GFP untagged Cx45 R184G-IRES-GFP and GFP tagged Cx45 R814G-GFP for functional and morphological studies respectively. As shown in Figure 7A,B, Cx45 R184G failed to form any functional GJ channels and were significantly different from cell pairs expressing WT Cx45 in both coupling% and coupling conductance (*G_j_*). When GFP tagged WT Cx45 (Cx45-GFP) was expressed in DKO HEK293 cells, clusters of GJ plaques were identified at the cell-cell junctions (Figure 7C, arrows), whereas R184G-GFP failed to form any clusters of GJ plaque-like structures at the cell-cell interface (Figure 7C).

**Figure 8.**
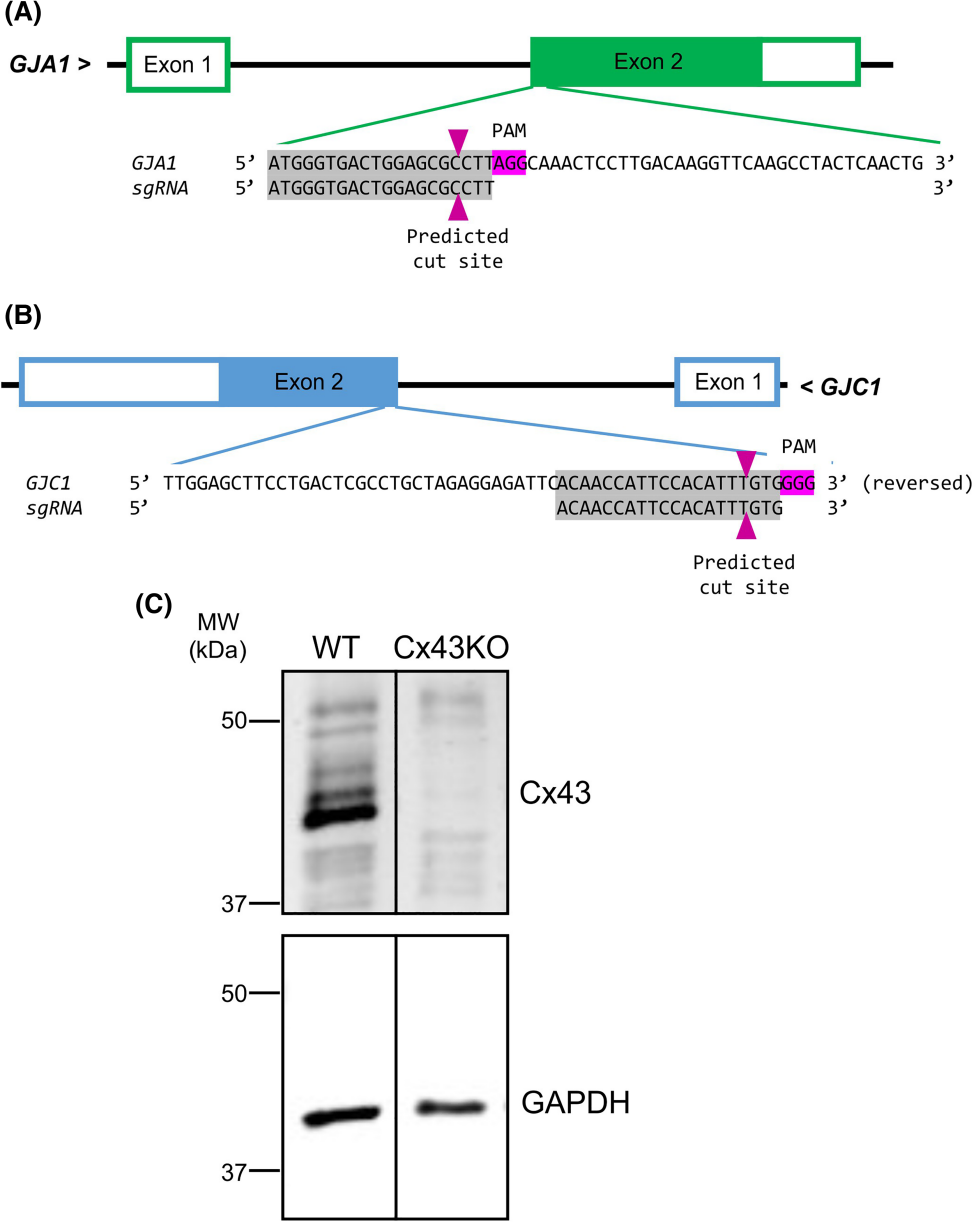
Design of sgRNA to knockout *GJA1* (encoding Cx43) and *GJC1* (encoding Cx45) genes in HEK293 cells. (**A**) and (**B**) panels illustrate the gene structure of *GJA1* and *GJC1* and the designed single guide RNA (sgRNA) targeting at the beginning of the coding regions (filled green and blue bars) of these genes. (**C**) Western blot shows that Cx43 is expressed and showed typical multiple bands in wildtype (WT) HEK293 cells. CRISPR-Cas9 gene ablation targeting the *GJA1* gene (encoding Cx43) was successful in deleting Cx43 (Cx43KO) in this clone of HEK293 cells. GAPDH was used as a loading control.

## Discussion

In the present study we genetically engineered a commonly used human model cell line, HEK293 cells, to be connexin deficient by ablating the endogenous Cx43 and Cx45 (the double knockout or DKO HEK293 cells). DKO HEK293 cells demonstrated excellent features for the functional study of GJs: (1) the DKO HEK 293 cells showed no GJ coupling (‘clean’ background) in multiple passages within the past few years; (2) expression of several exogenous human connexin genes individually, including Cx46, could successfully form functional GJs; (3) DKO HEK293 cells retained their accessibility for patch clamp analysis. Our dual patch clamp study on GJs formed by several human connexins (Cx46, Cx50, Cx45, Cx37, Cx36) demonstrated the feasibility of functional study of human GJs formed by these connexins, in the case of Cx46 and Cx26 it was possible to study at the single GJ channel level. Interestingly, the single Cx45 or Cx43 knock out HEK293 cells were found to be suitable for studying both macroscopic and single GJ channels on the endogenously expressed connexins. The genetically engineered HEK293 cells are excellent model cells to study human connexins either endogenously or exogenously expressed, including characterizing connexin variants linked to inherited diseases or those that exist in the general population [46]. A preliminary description of DKO HEK293 cells and successful application in studying human lens connexins was recently reported [47].

Mutations in more than 10 human connexin genes have been identified to possibly play a pathogenic role in inherited human diseases [7,8,48]. To ascertain whether these genetic variants play a pathogenic role in these diseases, one will need to have strong genetic evidence (e.g. co-segregation of disease with mutant carriers in multiple independent families), extremely low frequency or non-existence of the variant in the exomes of the general population (such as gnomAD) [49], and functional evidence of connexin mutants with altered function in either a cell model or an animal model [50]. For the functional studies on disease-linked connexin variants, each of the model systems showed some limitations as discussed below.

Mouse models of connexin variants are an excellent way to study human disease-linked variants and have been successful in recapitulating some aspects of the relevant human diseases [12,51,52]. The mouse models work especially well for those connexins that showed near identical protein sequences between mouse and human connexins (such as Cx32 and Cx43 with 99% and 97% sequence identity, respectively). But in cases where connexins have lower sequence identity between human and mouse, e.g. Cx46 (71% sequence identity) and Cx31.1 (77% sequence identity), it is not clear if one should use mouse or human connexin as template to generate the variant mouse model or whether the resultant mouse model would recapitulate key pathological manifestations of the human connexin diseases. It is also important to note that the connexin tissue distribution in mice is not always the same as those in humans. For example, mouse heart atrio-ventricular (AV) nodal cells express both Cx45 and Cx30.2 while in the human heart AV nodal cells only Cx45 is expressed without any expression of the human Cx31.9 (the orthologue of mouse Cx30.2) [53–55]. In this case, the vulnerability of action potential propagation in the human heart AV nodal cells cannot be recapitulated in the mouse models [56]. In addition, relatively high costs and longer time required for developing mouse models for connexin mutants are some additional hindering factors to consider when using mouse models for newly discovered connexin variants. Thus, many laboratories choose to use *in vitro* cell models to study the functional outcome of human disease-linked connexin mutants.

As discussed earlier the commonly used cell models to study expressed human connexins showed some significant limitations, including non-mammalian expression system (*Xenopus* oocytes expression studies) with different post-translational modifications, non-human cell context (such as N2A or RIN cells) with different inter-species interactomes especially those connexins with more variabilities between human and rodent, and in those human model cells (such as HeLa and SKHep1) they tend to have a higher level of heterogeneous background coupling or some less favorable properties for functional studies using patch clamp [39]. Our genetically engineered HEK293 cells overcome some of these limitations. The DKO HEK293 cells have been successfully used to study not only the properties of homotypic GJs, but also heterotypic docking compatibilities of human lens connexins, Cx46, Cx50, and Cx43 [47]. Our present study extended the use of DKO HEK to several other human connexins, and they were all expressed well and formed functional GJs with different signature gating properties.

Among all the human disease linked connexin mutants, the most prevalent cases are mutations in Cx26 linked sensorineural hearing loss [57]. It is interesting to note that some Cx26 mutants are very severe and not only associated with hearing loss but also with skin diseases known as syndromic hearing loss [58]. Here we showed that Cx26 was able to form functional GJ channels in DKO HEK293 cells and in some cases with only one or two channels between a cell pair providing an excellent vehicle to study mechanistic defects associated Cx26 mutants in individual GJ channels. Expression of a congenital heart disease and arrhythmia linked mutation in Cx45 (R184G) in DKO HEK293 cells resulted in no functional GJ formed, similar to that observed in N2A cells [45]. The failure to cluster at the cell-cell junctions of Cx45 R184G in DKO HEK293 cells was also observed in HeLa cells (Chen and Bai unpublished observations). These functional and morphological studies demonstrate that DKO HEK293 cells are an excellent model to study disease-linked mutants. We believe that the GJ channel properties obtained from this human cell line would be a much better reflection of their function in the human body than those obtained from *Xenopus* oocyte and/or rodent cell line expression systems. We have summarized some key features of commonly used expression vehicles on their strengths and weaknesses for functional study of GJ channels (Table 3).

**Table 3.** Comparison of model cells used for functional study of GJ channels

Model cells	Mammalian cells	Human cell context^§^	Coupling conductance measurements	Single channel recordings^‡^	Background GJ coupling^†^
*Xenopus* oocyte pairs	No	No	Yes	No	High
N2A cells	Yes	No	Yes	Yes	None or extremely low
HeLa cells	Yes	Yes	Yes	Yes	Moderate
RIN cells	Yes	No	Yes	Yes	Low
DKO HEK293 cells	Yes	Yes	Yes	Yes	None or extremely low

Notes: DKO HEK293, double knockout human embryonic kidney 293; ^§^some human connexins, like Cx46, does not express well in N2A cell, but human Cx46 expressed well in DKO HEK293 cells; ^‡^it is possible to perform single GJ channel current recording for some connexins; ^†^several factors could affect the background GJ coupling depending on the model cells, consistency of culture conditions, genetic stability of the model cells, etc. Built-in positive and negative controls should be routinely included to ensure the data obtained are not due to background GJs.

It is interesting to note that the single knockout of either Cx45 or Cx43 in HEK293 cells, yield a good level of endogenously expressed Cx43 or Cx45, respectively, for functional characterizations of GJs at both macroscopic and single GJ channel levels. This is a very convenient feature for studies on these human GJs, especially for Cx43 GJ which is extremely difficult to have optimized experimental conditions in recombinant overexpression model cells due to the following reasons. Depending on the connexins under study, the overexpression model cells tend to have a high level of expression of connexins because efficient promoters are commonly used in the expression cDNA vectors. Abundant connexin expression often ends up with highly GJ coupled cell pairs exceeding the ideal GJ coupling conductance range of under ∼10 nS to have proper voltage clamp with minimum voltage clamp errors [59,60]. When the coupling conductance (*G_j_*) is at ≥50 nS in a cell pair, then it is impossible to know for sure if the cell pair is highly GJ coupled or simply an apparent ‘cell pair’ not yet finished cell proliferation into two cells (known as cytoplasmic bridges, i.e. the apparent two cells in the ‘cell pair’ is still connected by their cytoplasm) which we encounter from time to time especially when studying recombinantly expressed Cx43. The data on these apparently highly GJ coupled cell pairs cannot be counted as GJ coupled pairs and must be discarded from our experimental data. To reduce the probability of having apparently highly GJ coupled cell pairs, we often design our experiments to capture the early phase of connexin expression by reducing the replating time, finding cell pairs with low and different levels of fluorescent reporters between the two cells in a pair (when using in frame fusion tagged or bicistronic expression vectors with GFP or RFP as reporter), which worked well in some experiments but not without negative consequences, such as reducing replating time could artificially increase the rate of false negative cell pairs (lowering the percentage of coupled cell pairs in our bar graphs for connexins fully capable to form GJs) and often technically very challenging as we can only patch on cells fully attached to the glass coverslips (usually require 30 min or longer). Different connexins appear to have somewhat different abilities in forming GJs between cell pairs and Cx43 is one of the easiest and earliest to form functional GJs and therefore the hardest to obtain optimized *G_j_* in cell pairs and virtually impossible to study single channel properties without introduction of GJ blockers or use acidifications as described in some earlier studies for Cx43 GJs or other connexin GJs [61,62]. While in the Cx45KO or Cx43KO HEK293 cells, we could find cell pairs with *G_j_* under 10 nS routinely and was feasible to obtain cell pairs with only 1–2 functional GJ channels with excellent signal noise ratio to perform single channel analysis without any introduction of GJ blockers. This is critical if one would like to study mechanisms of certain chemical modulations for these two cardiovascular GJ channels.

Genetically engineered HEK293 cells in our hand showed excellent genetic stability. The DKO HEK293 cells consistently show no GJ coupling over the last few years with multiple passages [47] and the single Cx43KO and Cx45KO also showed consistent GJ channel properties as those previously reported for Cx45 and Cx43 GJs [34,63]. We believe that these features of genetically engineered HEK293 cells are much better than those of HeLa or SKHep1 cells and will be used widely for characterizing human GJ channel properties using patch clamp and may also serve as a convenient model cell for dye-transfer studies and structural studies on recombinantly expressed human GJs.

In conclusion, we genetically engineered human HEK293 cells to either completely or partially delete the endogenously expressed Cx43 and Cx45. These cells showed excellent features for patch clamp analysis of human GJs and studying human disease-linked connexin variants in a human cell context will facilitate more accurate identification for pathogenicity of more and more identified disease-linked connexin variants.

## Materials and methods

### CRISPR-Cas9 gene ablation

A version of HEK293 cells were purchased from Agilent (known as AD-293 cells with catalog part# 240085). CRISPR-Cas9 was used to genetically ablate Cx43 as described [47,64]. Briefly, HEK293 cells were transiently transfected using Mirus TransIT-LT1 transfection reagent (MIR 2300, Mirus Bio, Madison, WI, U.S.A.) with the pSpCas9(BB)-2A-GFP plasmid (PX458, Addgene, Cambridge, MA, U.S.A.), which encodes for the Cas9 protein and a cloning background for the single guide RNA (sgRNA). The gRNAs used to target the exon of *GJA1* (human Cx43) were designed using the Sanger CRISPR finder (https://wge.stemcell.sanger.ac.uk/find_crisprs), and predicted to have low off-targets: 5′ ATGGGTGACTGGAGCGCCTT 3′ (Figure 8A). Following transfection, GFP-expressing cells were sorted using fluorescence activated cell sorting system at London Regional Flow Cytometry Facility and individual clones were examined for Cx43 ablation using Western blot (Figure 8C) and dual patch clamp method. Following similar procedures, Cx45 was also ablated on Cx43 knockout line as well as wildtype (WT) HEK293 cells with the following sgRNAs 5′ ACAACCATTCCACATTTGTG 3′ (Figure 8B). As no reliable antibody for Cx45 could be identified in our hands, we used dual patch clamp method to screen for successful knockout lines.

### Cell culture

WT, Cx43 knockout (Cx43KO), Cx45 knockout (Cx45KO), and double knockout (DKO with both Cx43 and Cx45 knockout) HEK293 cells were cultured in DMEM containing 4.5 g/l d-(+)-glucose, 584 mg/l l-glutamine (4 mM), 110 mg/l sodium pyruvate, 10% fetal bovine serum (FBS), 1% penicillin (100 units/ml), and 1% streptomycin (100 μg/ml or 172 μM), in an incubator with 5% CO_2_ at 37°C similar to those described earlier [65,66]. Prior to patch clamp recording, HEK293 cells were replated onto glass coverslips for different durations. For study on coupling percentage (coupling%) of cell pairs and coupling conductance (*G_j_*) of WT and Cx45KO the replating time was 0.7–1.5 h. For Cx43KO cells, a much longer replating time (18–20 h) was used to obtain data for coupling% and *G_j_*. For DKO cells both 0.7–1.5 and 18–20 h replating time were used. To increase the odds of observing single GJ channel current (*i_j_*s) in cell pairs, we shorten the replating time to 0.5–1 h for Cx45KO and Cx43KO HEK293 cells.

### Expression vectors and transfection

Human Cx46, Cx50, Cx45, and Cx37 were subcloned individually into a pIRES2-EGFP expression vector as described in our earlier studies [29,30,47]. Human Cx36 cDNA was obtained from R&D Systems (Cat# RDC1280) and was subcloned into pIRES2-EGFP expression vector between restriction enzyme sites NheI and EcoRI. All these human connexins were sequenced to ensure no errors in these expression vectors.

DKO HEK293 cells were transfected at a 1:2 ratio of cDNA : transfection reagent, with 0.6–1 μg of a cDNA construct and 1.2–2 μl of X-tremeGENE HP DNA transfection reagent (Roche Diagnostics GmbH, Cat#06366546001) in Opti-MEM + GlutaMAX-I medium (Cat#51985-034) for 5 h. Medium was changed back to FBS-containing DMEM after transfection until the following day, where transfected DKO HEK293 cells were replated onto glass coverslips. Coverslips were transferred to a recording chamber after 2–3 h, where isolated cell pairs successfully transfected with the connexins of interest together with the GFP as reporter were selected to study the formation of various homotypic GJs in our DKO HEK293 cells via dual whole cell patch clamp.

### Western blot

Soluble proteins were separated via SDS–PAGE and transferred to a 0.45 µm nitrocellulose membrane. The following primary antibodies were used for immunoblotting: rabbit anti Cx43 (C6219, Sigma–Aldrich); mouse anti GAPDH (MAB374, Chemicon). The following secondary antibodies were used: goat anti-rabbit IRDye 800 (611-132-122, Rockland) and goat anti-mouse Alexa Fluor 680 (A21076, ThermoFisher). The relative quantities of the immunoblotted proteins were normalized to GAPDH using the Odyssey Infrared Imaging System (Li-Cor Biosciences, Lincoln, NE, U.S.A.). As shown in Figure 8C, Cx43 was successfully deleted.

### Electrophysiological recording

Glass coverslips with HEK293 cells were placed into a recording chamber on an upright fluorescent microscope (BX51WI, Olympus). The chamber was filled with extracellular solution (ECS) at room temperature (21–24°C). The ECS contained (in mM): 135 NaCl, 2 CsCl, 2 CaCl_2_, 1 MgCl_2_, 1 BaCl_2_, 10 HEPES, 5 KCl, 5 d-(+)-glucose, 2 sodium pyruvate, pH 7.4 with 1 M NaOH, and with an osmolarity of 310–320 mOsm. Patch pipettes were pulled using a micropipette puller (PC-100, Narishige International USA Inc., Amityville, NY, U.S.A.) and filled with intracellular solution containing (in mM): 130 CsCl, 10 EGTA, 0.5 CaCl_2_, 2 Na_2_ATP, 3 MgATP, 10 HEPES, adjusted to pH 7.2 with 1 M CsOH, and with an osmolarity of 290–300 mOsm. Patch pipette resistance was in the range of 2–4 MΩ. Isolated HEK293 cell pairs were selected for dual whole cell voltage clamp experiment. Initially, both cells in a pair were voltage clamped at 0 mV, then one cell in the pair was stepped to a 20 mV voltage pulse of 7 s (pulsing cell) while the other cell of the pair was holding at a constant voltage of 0 mV (current recording cell). Transjunctional currents (*I_j_*s) were amplified with a MultiClamp 700A amplifier (Molecular Devices, Sunnyvale, CA, U.S.A.). *I_j_*s were low-pass filtered at 1 kHz (Bessel filter) and digitized via an AD/DA converter at a sampling frequency of 10 kHz (Digidata 1550, Molecular Devices) to a computer with pClamp 10.7 (Molecular Devices).

### Transjunctional voltage-dependent gating

To study transjunctional voltage dependent gating (*V_j_*-gating) properties in GJ coupled cell pairs, a series of voltage pulses (±20 to ±100 mV with 20 mV increment) were administrated to one of the cell pair to establish transjunctional voltage (*V_j_*). The other cell was held at 0 mV to record transjunctional current (*I_j_*). In experiments with Cx43KO HEK293 cells and recombinant expression of some human connexins, *V_j_* pulses of ±5 mV were also used because some GJs exhibited *V_j_*-gating at ±20 mV. In most cases, *I_j_* peaked at the beginning of a 7-s *V_j_* pulse, then declines to a steady state at the end (more pronounced with high *V_j_*s). The GJ coupling conductance (*G_j_*) was calculated by *G_j_* = *I_j_*/*V_j_* at *V_j_*s of ±20 mV for each engineered or WT HEK293 cell pair. Cell pairs that have *G_j_* >50 nS were excluded from coupling% analysis because it is impossible to differentiate the GJ-coupled cell pairs from ‘the apparently highly coupled cell pairs (due to cytoplasmic bridge)’. Only cell pairs that have *G_j_* lower than 9 nS were selected for *V_j_*-gating study to avoid voltage clamp errors [59,60]. The steady-state *G_j_* was normalized to the peak *G_j_* to obtain a normalized steady-state junctional conductance (*G_j_*_,*ss*_) for each *V_j_*. A *G_j_*_,*ss*_ - *V_j_* plot was then generated and fitted with a two-state Boltzmann equation for each *V_j_* polarity. The Boltzmann equation is:

$$G_{\;j,ss}=\frac{G_{\max}\;-\;G_{\min}}{1\;+\;\exp[A(V_{j}-V_{0})]}+G_{\min}$$

*V*_0_ is the *V_j_* at which the normalized steady-state conductance, *G_j_*_,*ss*_ = [(*G*_max_ - *G*_min_)/2 + *G*_min_]. *G*_max_ is the normalized maximum conductance, while *G*_min_ is the normalized minimum conductance. ‘*A*’ is the slope of the fitted curve that represents the *V_j_*-gating sensitivity [67].

### Single GJ channel current recording and analysis

Single channel currents (*i_j_*s) were observed in cell pairs with few active GJ channels. The recorded *i_j_*s were further filtered using a low-pass Gaussian filter at 200 Hz in Clampfit for measuring current amplitude and display in figures. The amplitude of *i_j_* values for the fully open state at different *V_j_* values were measured with fitting Gaussian functions on all point current amplitude histograms. The *i_j_*s of different cell pairs were averaged under the same *V_j_* regardless of *V_j_* polarity to generate *i_j_* – *V_j_* plot. Linear regression of *i_j_* – *V_j_* plot was used to obtain slope unitary conductance (*γ_j_*) as described earlier [66,68].

### Localization of Cx45 variant R184G

Fluorescent protein GFP was inframe fusion tagged at the carboxyl terminus of Cx45 variant R184G (R184G-GFP) similar to earlier studies [10,30]. R184G-GFP and WT Cx45-GFP were expressed individually in DKO HEK293 cells. Culture and transfection were the same as described earlier for functional studies. One day after transfection, cells were replated on glass bottom dises for 3–5 h to allow formation of morphological GJ plaques. Confocal microscope (Zeiss LSM800 with Airyscan, Zeiss, Oberkochen, Germany) with 40× water-immersion lens (NA1.2) was used to study the localization of Cx45-GFP and R184G-GFP. Bright green fluorescent clusters (deemed to be GJ plaques) could be observed at the cell-cell junctions of Cx45-GFP expressing cells, but no morphological GJ plaques were observed at cell-cell junctions in R184G-GFP expressing cells.

### Statistical analysis

The data on percentage of cell pairs coupled (coupling%) and coupling conductance (*G_j_*) bar graphs are expressed as median values and the error bars represent interquartile range (IQR). Kruskal–Wallis followed by Dunn's *post hoc* test was used to compare coupling% and *G_j_*. For analysis of *V_j_*-gatings of the Boltzmann fitting parameters for Cx43KO and Cx45KO HEK293 cells and *i_j_* – *V_j_* plots, data are expressed as mean ± SEM. An unpaired Student's *t*-test was used to compare Boltzmann parameters and single channel conductance (*γ_j_*). Statistical significance is indicated with the asterisks on the graphs (*P < 0.05; **P < 0.01; or ***P < 0.001).

## Data Availability

All data in this study are available from the corresponding author upon request.

## Abbreviations

Cx43: connexin43
Cx45: connexin45
Cx50: connexin50
ECS: extracellular solution
FBS: fetal bovine serum
GJ: gap junction
*G_j_*_,*ss*_: normalized steady-state junctional conductance
*G_j_*: gap junctional conductance
HEK: human embryonic kidney
IQR: interquartile range
*I_j_*: macroscopic junctional current
*i_j_*: single channel current
KO: knockout
RIN: rat insulinoma
sgRNA: single guide RNA
*V_j_*: transjunctional voltage
WT: wildtype
